# Neurotensin Receptor 1 Gene (*NTSR1*) Polymorphism Is Associated with Working Memory

**DOI:** 10.1371/journal.pone.0017365

**Published:** 2011-03-04

**Authors:** Jin Li, Chuansheng Chen, Chunhui Chen, Qinghua He, He Li, Jun Li, Robert K. Moyzis, Gui Xue, Qi Dong

**Affiliations:** 1 National Key Laboratory of Cognitive Neuroscience and Learning, Beijing Normal University, Beijing, People's Republic of China; 2 Department of Psychology and Social Behavior, University of California Irvine, Irvine, California, United States of America; 3 Department of Psychology, University of Southern California, Los Angeles, California, United States of America; 4 Department of Biological Chemistry, University of California Irvine, Irvine, California, United States of America; Alexander Flemming Biomedical Sciences Research Center, Greece

## Abstract

**Background:**

Recent molecular genetics studies showed significant associations between dopamine-related genes (including genes for dopamine receptors, transporters, and degradation) and working memory, but little is known about the role of genes for dopamine modulation, such as those related to neurotensin (NT), in working memory. A recent animal study has suggested that NT antagonist administration impaired working memory in a learning task. The current study examined associations between NT genes and working memory among humans.

**Methods:**

Four hundred and sixty healthy undergraduate students were assessed with a 2-back working memory paradigm. 5 SNPs in the *NTSR1* gene were genotyped. 5 ANOVA tests were conducted to examine whether and how working memory differed by NTSR1 genotype, with each SNP variant as the independent variable and the average accuracy on the working memory task as the dependent variable.

**Results:**

ANOVA results suggested that two SNPs in the *NTSR1* gene (rs4334545 and rs6090453) were significantly associated with working memory. These results survived corrections for multiple comparisons.

**Conclusions:**

Our results demonstrated that *NTSR1* SNP polymorphisms were significantly associated with variance in working memory performance among healthy adults. This result extended previous rodent studies showing that the NT deficiency impairs the working memory function. Future research should replicate our findings and extend to an examination of other dopamine modulators.

## Introduction

Working memory (WM) refers to the brain function involved in the temporary manipulation (i.e., processing, integration, storage, retrieval, etc.) of the information necessary for complex cognitive tasks such as language comprehension, learning, and reasoning [1]. Twin studies have shown that individual differences in WM capacity are likely to have a significant genetic basis, with the estimated heritability between 33% and 64% [2], [3], [4]. Pharmacological studies on both animals [5] and humans [6] have suggested that dopamine (DA) in the brain modulates WM capacity. Finally, molecular genetics studies showed significant associations between DA-related genes (such as COMT, DRD4, and DAT) and WM [7], [8]. However, these studies only focused on genes for DA receptors, transporters, and degradation, it is not clear if there are any associations between genes that modulate DA and WM.

Neurotensin (NT), a tridecaptide widely distributed throughout the central nervous system [9], acts as a neuromodulator, particularly of DA transmission in several areas in the brain, such as nigrostriatal and mesolimbic pathways [10]. During the past decades, NT was widely studied with regard to its interaction with the central DA system. Animal studies suggested that NT is co-localized with DA in a subset of dopaminergic neurons projecting from the ventral tegmental area to the medial prefrontal cortex (PFC) [11], [12]. The anatomical overlaps between the NT and the DA systems allow them to have functional interactions at cellular level [13]. For example, NT can activate dopaminergic neuron firing [14], [15]. Local administration of NT in the PFC has also been found to increases DA release [16]. In addition, rodent studies have shown that NT or NT analog can enhance DA release in the striatum, nucleus accumbens, and PFC [17], [18]. Furthermore, these cellular interactions have behavioral consequences. For example, administration of NT or NT analogue can stimulate DA-dependent behaviors, while NT antagonist can block the effect of NT analogue [19], [20]. These results suggest a possibility that NT, which modulates DA transmission (e.g., in the PFC), would also regulate DA-dependent (and PFC-related) WM capacity.

Human neurotensin receptor 1(NTSR1, coded by the human gene *NTSR1*, which is located on chromosome 20q13) is a high affinity NT receptor with 7 transmembrane spanning regions and corresponds to G-protein-coupled receptor [21], [22]. NT, acting on NTSR1, reduces the physiological function of D2 dopamine receptor [14]. In addition, a recent study showed altered DA receptor mRNA expression in NTSR1 null mice [23]. Rodent studies suggest that NT receptors are located on dopaminergic cell bodies in both nigrostriatal and mesolimbic systems [24]. MRNA expression [25] and protein content [26] of NTSR1 are distributed throughout the central nervous system including the PFC and the anterior cingulate, two critical regions for working memory [27], [28]. One recent rodent study has suggested that administration of NTSR1 antagonist impairs working memory in a learning task [29]. However, no studies have been done with humans about potential associations between *NTSR1* gene variants and WM capacity.

Some indirect evidence for such associations came from research on the association between NT and schizophrenia. Because impaired working memory has been found to be an endophenotype of schizophrenia [30], [31], susceptibility genes for schizophrenia may be related to WM. Previous studies have shown an important role for NT and NTSR1 in schizophrenia. The density of NT receptors is decreased in schizophrenia patients [32]. Furthermore, cerebrospinal fluid (CSF) levels of NT are inversely related to the severity of psychosis in untreated schizophrenics, whereas increased levels of NT are associated with improvement in symptoms during treatment [33]. Similarly, a study with rats confirmed the role of NT neurotransmission in the effectiveness of antipsychotic drugs [34]. In terms of the *NTSR1* gene, one previous study [35] reported that *NTSR1* gene polymorphism was associated with schizophrenia, although two later studies did not replicate this result [36], [37]. Possible explanations for the disagreement could be variations in genotype frequencies across populations and selection of different loci for the different studies. Future research needs to include multiple SNPs.

The present study investigated whether genetic variants in the *NTSR1* gene were associated with performance on a WM task in a large Chinese sample. We included five SNPs in the *NTSR1* gene.

## Results

The mean accuracy of all 458 subjects on the WM task was 0.85 (SD = 0.08). Males and females had comparable mean accuracy, 0.85(SD = 0.08) and 0.86(SD = 0.08), respectively, *F* (1,456) = 1.22, *p* = 0.27.

Allele frequencies of the five SNPs in our sample were similar to those of Han Chinese in the Hapmap database (www.hapmap.org). There were no gender differences in allele frequencies for all five SNPs (all *t*s<0.4, all *ps*>0.05).

**Figure 1:**
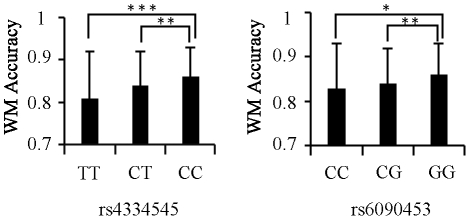
Associations between SNP polymophisms (rs4334545 and rs6090453) and WM performance. Working memory performance was compared across different genotypes by one-way ANOVA followed by Fisher's LSD *post hoc* test. * *p*<.05, ** *p*<.01, ***p<.001.

ANOVA results showed that two of the five SNPs (rs4334545 and rs6090453) significantly modulated WM performance (for rs4334545, *F* (2,455) = 7.82, *p* = 4.6×10^−4^; for rs6090453, *F* (2,455) = 5.63, *p* = 3.8×10^−3^, both of which survived Bonferroni corrections. See **Table 1**). Fisher's least significant difference (LSD) *post hoc* tests showed that, for rs4334545, WM performance was significantly higher in CC genotype than in CT or TT genotype (*p* = 7.0×10^−3^ and *p* = 9.4×10^−4^, respectively); for rs6090453, GG genotype showed significantly higher WM performance than did CG genotpe (*p* = 2.8×10^−3^), but the difference between GG and CC genotypes (*p* = 0.029, Figure 1) was no longer significant after Bonferroni correction (which set the alpha at .01, see the method section). Results were similar when we combined the homozygotes of minor allele and the heterozygotes together (for rs4334545, *F* (1,456) = 11.84, *p* = 6.3×10^−4^; for rs6090453, *F* (1,456) = 10.97, *p* = 1.0×10^−3^).

**Table 1:** Information of 5 SNPs on NTSR1 and their effects on WM performance.

SNP	Position on chr 20	Genotype	Counts	Frequency	WM performance	F (2, 455)	p
rs2427399	60801890	AA	24	0.05	0.86 (0.07)	1.47	0.23
		AG	159	0.35	0.84 (0.08)		
		GG	275	0.60	0.86 (0.07)		
rs6062460	60820535	TT	1	0.00	0.85	0.14	0.87
		CT	50	0.11	0.86 (0.06)		
		CC	407	0.89	0.85 (0.08)		
rs4334545	60823622	TT	27	0.06	0.81 (0.11)	7.82***	4.6×10^−4^
		CT	160	0.35	0.84 (0.08)		
		CC	271	0.59	0.86 (0.07)		
rs6090453	60825807	CC	35	0.08	0.83 (0.10)	5.63**	3.8×10^−3^
		CG	191	0.41	0.84 (0.08)		
		GG	232	0.51	0.86 (0.07)		
rs6089784	60870007	TT	23	0.05	0.86 (0.07)	0.56	0.58
		CT	167	0.36	0.86 (0.07)		
		CC	268	0.59	0.85 (0.08)		

**p<.01.

***p<.001.

**Figure 2:**
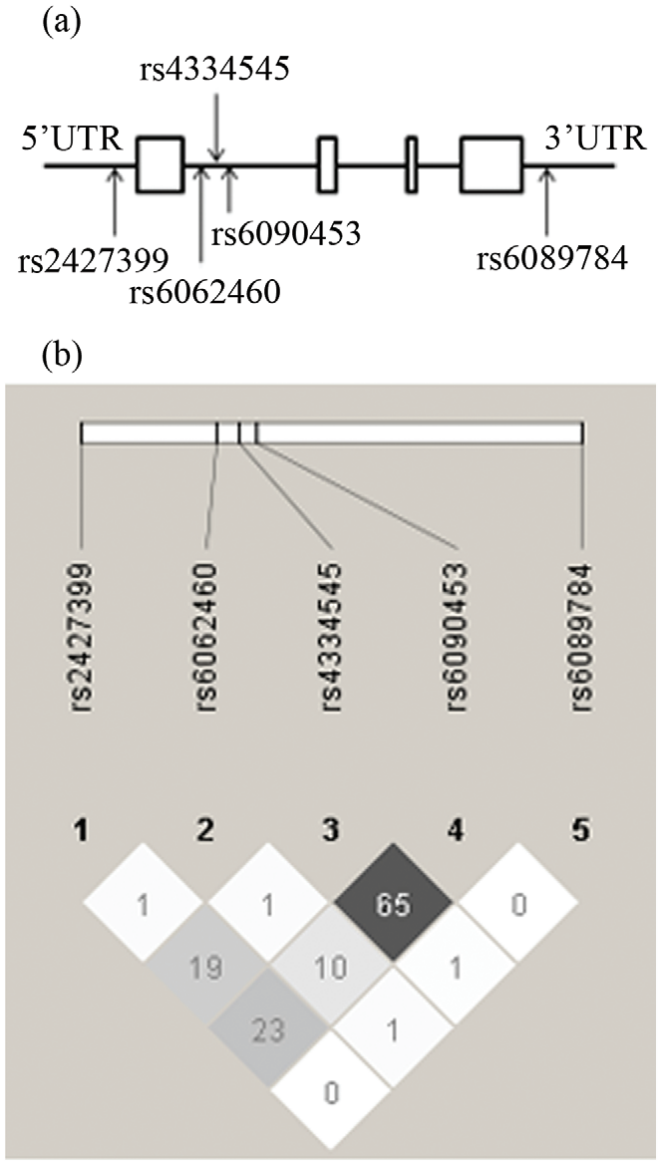
Schematic representation of the *NTSR1* gene and linkage disequilibrium map of selected SNPs. (a), Schematic representation of *NTSR1* gene, and relative positions of the 5 selected SNPs. The *NTSR1* gene is comprised of 4 exons and 3 introns. Boxes represent exons. (b), Linkage disequilibrium map using data from our study. Pairwise linkage disequilibrium values (r^2^ values) are indicated. White, shades of gray, and black squares indicate no LD (r^2^ = 0), intermediate LD (0<r^2^<1), and strong LD (r^2^ = 1), respectively.

The linkage disequilibrium (LD) between SNPs in the *NTSR1* gene was calculated by Haploview [38]. The LD calculation result is shown in Figure 2, r^2^ between rs4334545 and rs6090453 was 0.65. Our LD result was similar to that in the Han Chinese (CHB) sample from the HapMap database (www.hapmap.org). No SNP was blocked into a haplotype in these five SNPs; therefore we did not perform any further haplotype association tests.

**Figure 3:**
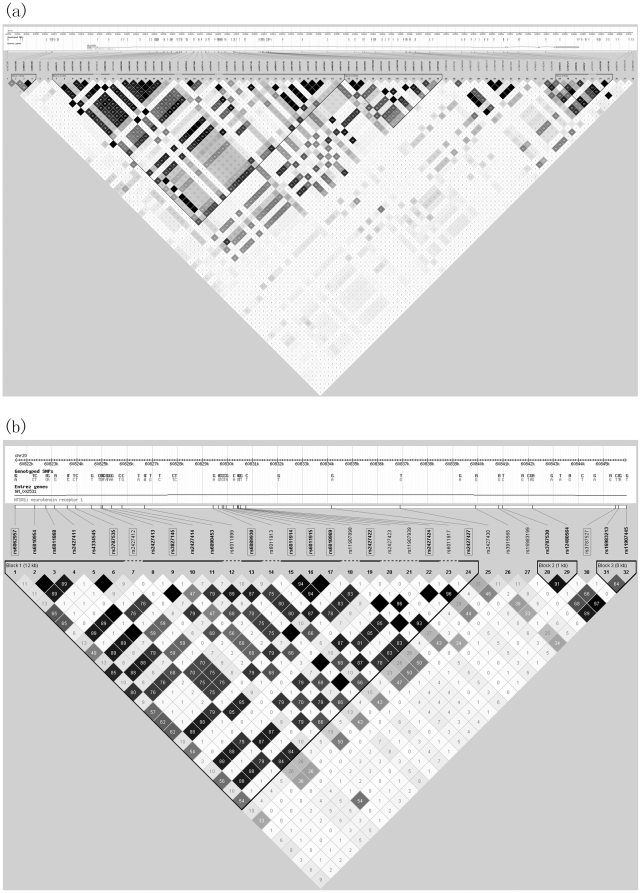
Linkage disequilibrium plot. (a), Plot of 68 kb genomic region for imputed SNPs region. Positions of genes, SNPs genotyped in the HapMap, and linkage disequilibrium among SNPs (r^2)^ are shown. The plot was generated with Haploview (release 21/phase II Jan 06, NCBI B35 assembly, CHB+JPT sample, chr20 start and end kb: 60802–60871). Imputation analysis illustrates that the strongest association signals are localized to a 24 kb region (chr20 start and end kb: 60821–60846) shown in detail in (b). Imputed SNPs that were statistically associated with working memory (p<1.2×10^−3^) are boxed.

Imputation of SNPs from our data and the HapMap II data for the selected 68 kb region for 458 participants allowed us to assess whether additional association signals might be present in this region. On the basis of these imputed data, the strongest evidence for association was found for SNP rs3787527 (*p* = 1.73×10^−22^; Figure 3 and table 2). Indeed, within this 68 kb region, the strongest association signals from imputed data were localized to a 24 kb region containing 12 highly correlated SNPs that are in intron1 of the *NTSR1* gene (table 2). Using data from the HapMap Project, we found that all the SNPs with statistical associations with WM performance except 2 SNPs (rs2427412 and rs3787527) were in the same haplotype block(12 kb length) with rs4334545 and rs6090453 (Figure 3). However, in view of the low minor allele frequency (MAF) of rs2427412 and rs3758527 (0.03 and 0.05, respectively), we need to be cautious about drawing conclusions regarding associations between these latter 2 SNPs and WM performance.

**Table 2:** Imputed SNPs showing statistical associations (p<0.05) with working memory performance.

SNP	Position on chr 20	Major allele	Minor allele	MAF^a^	p value	Beta	SE	Quality^b^
rs6062957	60821530	A	G	0.29	6.2×10^−04^	−0.25	0.07	0.99
rs3787535	60823966	G	A	0.23	1.1×10^−04^	0.30	0.08	1.00
rs2427412	60824580	G	T	0.03	4.9×10^−15^	−2.45	0.25	0.95
rs3827145	60825027	C	T	0.19	5.5×10^−05^	−0.41	0.10	0.91
rs6089930	60829446	A	G	0.29	6.3×10^−04^	−0.26	0.08	0.95
rs6011914	60829796	G	C	0.27	4.6×10^−04^	0.28	0.08	0.93
rs6011915	60829866	T	C	0.26	4.7×10^−04^	0.27	0.08	0.95
rs6010969	60829964	A	G	0.26	4.6×10^−04^	−0.27	0.08	0.95
rs2427422	60830418	G	A	0.24	1.9×10^−04^	0.30	0.08	0.97
rs2427424	60830533	G	T	0.24	1.9×10^−04^	−0.29	0.08	0.98
rs2427427	60834167	G	A	0.23	1.8×10^−04^	0.31	0.08	0.97
rs3787527	60845495	G	A	0.05	1.7×10^−22^	2.15	0.14	0.90

a MAF: minor allele frequency.

b Quality: the average posterior probability for the most likely genotype.

## Discussion

The present study used a relatively large Chinese sample to investigate if there were any associations between variants of the *NTSR1* gene and working memory (WM). We found that two SNPs' polymorphisms (rs4334545 and rs6090453) in the *NTSR1* gene were significantly associated with WM performance, suggesting that the *NTSR1* gene is involved in human higher cognitive function. Specifically, we found that C allele in rs4334545 and G allele in rs6090453 showed higher WM capacity than their counterparts. To our knowledge, this is the first study exploring the relationship between the *NTSR1* gene and WM in human subjects.

Several lines of previous research have suggested that dopamine neurotransmission plays a pivotal role in WM. First, pharmacological modulation of dopamine receptor signaling pathway can produce changes in functional circuits underlying WM in monkeys [39]. Second, human studies also showed that performance improvement after WM training was associated with changes in cortical dopamine D1 receptor binding [40]. Third, several DA system gene polymorphisms have shown to be associated with WM performance, including genes for dopamine receptors, transporters, and degradation. Taken together the results of our study and those of previous studies, it seems that selected genes for all important components of the DA system (DA receptors, transporters, and degradations, and now modulators) are associated with WM. The *NTSR1* gene encodes a high-affinity neurotensin receptor, namely NTSR1. *NTSR1* gene variants can alter neurotensin function in the central nervous system, which in turn modulates the DA system serving WM functions [41]. Neurotensin is widely distributed in dopaminergic neurons, and is a well-demonstrated modulator of dopamine transmission. Microinjection of neurotensin into the PFC has been found to alter dopamine cell firing and DA release [16], [42]. In addition, the effect of NT on DA release in the PFC was regulated by NTSR1 receptors [16]. The important role of NT in the DA system results in potential associations between NT and DA-related cognition. For example, NT agonist enhances memory consolidation through the DA system [43]. Another study also showed that NT deficiency impairs WM function. Rats infused with SR 48692, a preferential NTSR1 antagonist, showed more WM errors than those injected with saline [29]. Our study extends these results to human subjects.

The present study can also potentially offer some insights to the diagnosis and/or treatment of schizophrenia. WM deficit is widely considered as an endophenotype of schizophrenia [30], [31], and previous studies had already showed an important role of NT in schizophrenia. Our results suggested that the *NTSR1* gene was associated with this significant endophenotype of schizophrenia, and raised the possibility that we can take into account of this information for schizophrenia diagnosis. However, our study was limited to normal subjects, and future studies are needed to explore this possibility in schizophrenia subjects.

Two main limitations of the present study need to be discussed. First, our results are associative. We did not explore biochemical effects of the two SNP variants on the function of NTSR1 protein. These two SNPs (rs4334545 and rs6090453) are both in intron areas of the *NTSR1* gene, which may or may not have functional consequence on the protein. On the one hand, previous papers showed that intron variants may play important roles in gene expression [44], mRNA secondary structure formation [45], and transcriptional suppression of the genes [46]. It is possible that the 2 intronic SNPs associated with WM performance play a role in regulating *NTSR1* gene expression. Further genetic and biochemical studies are needed to test this hypothesis. Another possibility is that these 2 SNPs might have very high linkage disequilibrium with a mutation of a nearby SNP in exons. By conducting imputation analysis with the HapMap data, we found 12 imputed SNPs with significant statistical associations with WM performance, all of which were localized around these 2 SNPs and to a 24 kb region in intron1 of the *NTSR1* gene. The HapMap data further showed that 10 of the 12 SNPs were in the same 12 kb haplotype block with the 2 SNPs found in our study, which to some extent explained similar gene-behavior relationships of the *NTSR1* gene and WM using either the imputed SNPs or the 2 SNPs in our data. Given that none of the SNPs in the imputed association analyses from the HapMap data was in exons of the *NTSR1* gene, the current imputation analysis did not help us to find causative exon SNPs with WM performance. Future studies should explore the causative locus of the variants of NTSR1 function, and how the *NTSR1* gene variants impact on NTSR1 function and modulate dopamine.

The second limitation of our study is that we only examined one ethnic group (i.e., Han Chinese college students), which, from a genetic standpoint, has major advantages. However, the same strength of the current study limits the generalization of our results to other populations. Previous studies showed that gene-behavior relationship is modulated by sex, ethnicity and/or culture [47], [48] and further studies should be done to see if our results can be applied to other groups.

In summary, *NTSR1* SNP polymorphisms were significantly associated with variance in working memory performance in a sample of Chinese college students. This result extended a previous rodent study showing that NT deficiency impairs working memory [29]. Future research should replicate our findings in independent samples and extend to an examination of other dopamine modulators.

## Materials and Methods

### Participants

Four hundred and sixty (261 females) healthy undergraduate students (aged from 18 to 23 years, with a mean of 20.4 years, SD = 0.87) were recruited from Beijing Normal University. All participants were Han Chinese with normal or corrected-to-normal vision and without neurological or psychiatric history based on self-report. This study was approved by the Beijing Normal University's Institutional Review Board. Written informed consent was obtained from each participant.

### Genotype analysis

A 4 ml venous blood sample was collected from each participant. After blood samples were collected, genomic DNA was extracted according to the standard method within 2 weeks.

All samples were genotyped using the standard Illumina GoldenGate Genotyping protocol (see www.southgene.com.cn for details). Sixty genes (384 SNPs) involved in neurotransmitter system were typed in the gene-brain-behavior project. For each gene, several tag SNPs were selected based on the HapMap data (www.hapmap.org) to cover these genes using relatively few SNPs. Of interest to this study, 5 SNPs in *NTSR1* gene were selected, including rs2427399, rs6062460, rs4334545, rs6090453 and rs6089784. Samples with more than 10% missing calls were removed (n = 2, including 1 female), resulting in a final sample of 458 for subsequent analyses. All five *NTSR1* SNPs passed the criteria of a call rate of >90%, Minor Allele Frequency (MAF) of >0.05, and Hardy-Weinberger equilibrium (HWE) of *p*>0.05.

### WM tasks

WM was assessed with a 2-back WM paradigm [49]. Viewing a series of characters that were presented sequentially, participants performed three continuous judgment tasks: semantic judgment (whether the Chinese character on the screen was from the same semantic category as the character presented two characters earlier), phonological judgment (whether the current Chinese character rhymed with the one shown two characters earlier), and morphemic judgment (whether the current Tibetan letter was the same as the one presented two letters earlier). Participants did not know Tibetan letters. Each judgment task consisted of four blocks (10 trials each). Before the judgment tasks, participants had a practice block (judging small circles and squares), in which they had to pass 70% of trials before they could take the formal tests. Cronbach alpha in this sample was .82. The average score (accuracy) of the three WM tasks was used as the index of working memory in the current study [50].

### Data Analysis

Genotype was encoded as minor allele SNP-dosage (homozygote of major allele  = 0, heterozygote  = 1, homozygote of minor allele  = 2). In a preliminary analysis, five separate two-way ANOVAs were conducted to test the effects of gender, each SNP, and their interaction on WM task performance. Results showed that the main effects of gender were non-significant (all *p*s>0.05), so we excluded gender as an independent factor in the final ANOVA analysis. Finally, five ANOVAs were conducted separately for each SNP to test the main effect of each SNP. To correct for multiple comparisons, the statistical significance level of these ANOVAs was set as p<0.01 (0.05/5[SNP variants], i.e., Bonferroni correction for family-wise error). For those SNPs that passed the significance level, Fisher's least significant difference (LSD) *post hoc* tests (t-tests) were carried out to identify the direction of each effect. The *post hoc* tests were also corrected for multiple comparisons using the Bonforroni method. All statistical calculations were carried out in SPSS for windows (Release 15.0).

As an additional exploratory analysis, genotypes were imputed for all ungenotyped SNPs within candidate SNP regions in our study (68 kb, covering the *NTSR1* gene), using IMPUTE2[51], [52](https://mathgen.stats.ox.ac.uk/impute/impute_v2.html), based on the information from the five directly typed SNPs and the HapMap database (HapMap Data Rel 21/phase II, population: Japanese/Chinese). For these imputed data, association analysis was done with SNPTEST2 [53], [54](http://www.stats.ox.ac.uk/~marchini/software/gwas/snptest.html).

Only SNPs with a MAF larger than 1% and with a posterior-probability score of 0.90 or higher were considered for these imputed association analyses [52], [54]. Statistical significance level was defined as p<1.2×10^−3^ (0.05/43[imputed SNPs]) to adjust for multiple comparisons.
